# Draft Genome Sequence of the First Human Isolate of the Ruminant Pathogen *Mycoplasma capricolum* subsp. *capricolum*

**DOI:** 10.1128/genomeA.00583-15

**Published:** 2015-06-18

**Authors:** Frederik Valeur Seersholm, Anne Fischer, Martin Heller, Joerg Jores, Konrad Sachse, Tobias Mourier, Anders Johannes Hansen

**Affiliations:** a Centre for GeoGenetics, Natural History Museum of Denmark, University of Copenhagen, Copenhagen, Denmark; b International Livestock Research Institute, Nairobi, Kenya; c International Centre of Insect Physiology and Ecology, Nairobi, Kenya; d Institute of Molecular Pathogenesis, Friedrich-Loeffler-Institut, Jena, Germany; e Institute of Veterinary Bacteriology, University of Bern, Bern, Switzerland

## Abstract

*Mycoplasma capricolum* subsp. *capricolum* is a well-known pathogen of small ruminants. A recent human case of septicemia involving this agent raised the question of its potential pathogenicity to humans. We present the first draft genome sequence of a human *Mycoplasma capricolum* subsp. *capricolum* isolate.

## GENOME ANNOUNCEMENT

*Mycoplasma capricolum* subsp. *capricolum* is a known etiologic agent of contagious agalactia in small ruminants, a disease associated with chronic inflammation, arthritis, and mastitis (1). Even though *M. capricolum* subsp. *capricolum* is considered one of the least pathogenic members of the *Mycoplasma mycoides* cluster, disease outbreaks caused by this agent can have a significant impact on goat farming industries due to loss of milk production and increased mortality (2, 3). While *M. capricolum* subsp. *capricolum* infections are well known in sheep and goats, reports of other animal hosts are scarce (4). Recently, the *Mycoplasma capricolum* subsp. *capricolum* strain 14DL0024 was isolated from a hospitalized human displaying symptoms of septicemia (M. Heller, R. Schwarz, G. Noe, J. Jores, A. Fischer, E. Schubert, and K. Sachse, submitted for publication). Here, we report the draft genome sequence of the human *Mycoplasma capricolum* subsp. *capricolum* strain 14DL0024.

Genomic DNA of the bacterial isolate was extracted as reported elsewhere (A. Fischer, I. Santana-Cruz, E. Schieck, H. Gourlé, M. Lambert, H. W. Suvarna Nadendla, R. A. Miller, J. Hegerman, J. Meens, S. Vashee, J. Frey, and J. Jores, submitted for publication). DNA libraries were built using the NEBNext library kit E6070 and sequenced on the Illumina HiSeq 2000 platform, yielding a total of 20,483,838 paired-end reads. Reads were processed, trimmed, and assembled using AdapterRemoval (v1.1) (5), Novobarcode Beta-0.8, and Ray (2.3.1) (6). Lastly, contigs were extended and scaffolded by SSPACE basic (v.2.0) (7), yielding a set of scaffolds of which sequences shorter than 2 kilobases (kb) were discarded. The resulting draft genome yielded 7 scaffolds between 11 and 218 kb with a GC content of 23.7%, an *N*_50_ of 197,640 bp, and an *N*_90_ of 120,063 bp. With a total size of 964,668 nucleotides, the draft genome covers 95.5% of the length of the reference genome (GI:83319253) of 1,010,023 bp, indicating a high level of completion for the assembly.

To verify the species of the isolated bacteria, average nucleotide identity (ANI) analyses (8) were carried out, comparing the draft genome with available genomes of the *Mycoplasma mycoides* cluster. As expected, the analysis revealed the highest average nucleotide identity (97.77%) with *Mycoplasma capricolum* subsp. *capricolum* (GI:83319253), while the closely related *Mycoplasma capricolum* subsp. *capripneumoniae* strains M1601 (GI:326314730), ILRI181 (GI: 677282260), and F38 (GI:675241189) displayed slightly lower ANI values between 96.15% and 96.16%. The remaining members of the *Mycoplasma mycoides* cluster all exhibited significantly smaller ANI values below 93%, thus confirming the species of the draft genome to be *Mycoplasma capricolum* subsp. *capricolum*.

The draft genome was annotated by RAST (9), which identified 773 protein coding genes, 30 tRNAs, and 7 rRNAs. Fourteen genes were unique to the draft genome compared to the *M. capricolum* subsp. *capricolum* reference. Most notable is the presence of three key genes of type I restriction modification (RM) systems. Encoding the specificity (S), modification (M), and restriction (R) subunits, the three genes comprise the entire genetic basis for type I restriction modification. Considering the absence of RM genes in the reference genome and previous reports linking RM systems with bacterial virulence and immune evasion (10), this might indicate a role of the RM system in human *M. capricolum* subsp. *capricolum* infection. However, additional studies will be needed to support this claim.

### Nucleotide sequence accession numbers. 

This whole-genome shotgun project has been deposited at DDBJ/EMBL/GenBank under the accession no. LBMF00000000. The version described in this paper is the first version, LBMF01000000.
